# Electrically conducting films prepared from graphite and lignin in pure water

**DOI:** 10.3389/fbioe.2022.1049123

**Published:** 2022-11-08

**Authors:** Asami Suzuki, Yuichiro Otsuka, Kazuhiro Shikinaka

**Affiliations:** ^1^ Research Institute for Chemical Process Technology, National Institute of Advanced Industrial Science and Technology, Sendai, Japan; ^2^ Forestry and Forest Products Research Institute, Tsukuba, Japan

**Keywords:** Plant, biomass, lignin, graphite, electrically conducting material

## Abstract

In this study, we present electrically conducting self-standing graphite films consisting of lignin derivatives extracted by simultaneous enzymatic saccharification and comminution (SESC). Sonication of graphite powder in the presence of SESC lignin and pure water allows dispersion of the SESC-lignin-attached graphite without addition of other chemicals. The SESC-lignin-attached graphite having a diameter of several micrometers can be used as a surface electroconductive coating and molded into self-standing films by drying. The SESC-lignin-attached graphite film exhibits higher conductivity (∼2,075 S/cm) than graphite-based composites consisting of ordinary lignin derivatives. Manufacturing self-standing films of micrometer-sized graphite using SESC lignin enables high electrical conductivity of the SESC-lignin-attached graphite film. The size of the SESC-lignin-attached graphite is proportional to the conductivity of the film. The SESC-lignin-attached graphite also acts as an antiplasticizer and a conductive filler for polymer films, i.e., conductive films consisting of poly(ethylene glycol) or Li^+^ montmorillonite can be obtained through a water-based process.

## Introduction

Breaking away from oil-refinery-based industries is needed to realize a low-carbon society for sustainable development (Kamm et al., 2006). Plants are promising alternatives to petroleum-based resources and are expected to support biomass-refinery-based industries (Ragauskas et al., 2006; Ioelovich, 2015). Lignin consists of propenyl phenol units and is the second most-abundant nonedible plant biomass that has potential as a renewable alternative to synthetic aromatic polymers (Wang et al., 2019a,b; Huang et al., 2019). Because of its structural characteristics, lignin has been studied for material utilization, such as carbon fibers (Sun et al., 2022), flocculants (Wang et al., 2020), and composites (Chen et al., 2022). However, one important problem with the utilization of lignin derivatives is the necessity for hazardous chemicals in the extraction processes, such as pulping methods, which incur an environmental load and may cause deterioration of lignin during extraction (Adler, 1977; Aziz and Sarkanen, 1989; Shikinaka et al., 2010).

To overcome this problem, we recently proposed a novel lignin extraction method called simultaneous enzymatic saccharification and comminution (SESC), in which wet-type ultrafine bead milling and enzymatic reactions for plants are used to isolate polysaccharides and lignin, which are the main components of plants, in the form of sugar solution and lignin water dispersion from plants without the utilization of and contamination by toxic reagents and byproducts (Shikinaka et al., 2016; Navarro et al., 2018). The obtained sugar solution and lignin water dispersion can be applied to methane gas (Navarro et al., 2020), drinkable alcohol (Otsuka et al., 2020), and functional polymeric materials, such as heat-proofing fillers (Shikinaka et al., 2018a; Sotome et al., 2020; Shikinaka et al., 2021) and UV absorbers (Shikinaka et al., 2019; Shikinaka et al., 2020). The lignin derivatives extracted by SESC (later denoted as SESC lignin) are water-dispersed nanoparticles (Shikinaka et al., 2016).

In this study, we prepared self-standing graphite films consisting of SESC lignin to prepare biomass-based electroconductive materials. Graphite is one of the inexpensive carbon-based conducting materials. Hence, graphite electrodes are commonly used in various cells and batteries (Doeff et al., 1993; Chaudhuri and Lovely, 2003; Liu et al., 2004; Zhou et al., 2011; Sun et al., 2016; Xu et al., 2019). Lignin derivatives have been used as graphite dispersants in solvents (Winter and Besenhard, 1999; Yang et al., 2010; Yang et al., 2014); however, ordinary lignin derivatives may contain impurities owing to the reaction of lignin with sulfides in the extraction procedure, especially the pulping process (Sarkanen and Ludwig, 1971; Kai et al., 2016). In contrast, owing to the absence of sulfides in the SESC-based lignin extraction process (Adler, 1977), SESC lignin is free of impurities, such as sulfur content. Furthermore, water-dispersed SESC lignin has an anionic charge, which encourages it to act as a dispersant of graphite materials such as lignin sulfonate (Liu et al., 2015). Thus, the combination of graphite and SESC lignin enables production of lignin-based carbon materials without impurities.

Herein, we estimated the function of SESC lignin as a dispersant for graphite. Attaching the SESC lignin to graphite and further miniaturization by sonication in pure water produced water-dispersed lignin-attached graphite (LAG) flakes with an average diameter of several micrometers, i.e., SESC lignin acts as a dispersant of graphite that is free from toxic additives such as surfactants and organic solvents. Casting and drying LAG water dispersion on an insulated substrate resulted in an electroconductive surface, i.e., LAG dispersion can be treated as an electroconductive ink. Self-standing conductive graphite films can be prepared only by drying the LAG water dispersion; here, the LAG-based films exhibit a higher conductivity (400–2,000 S/cm) than graphene-based composites consisting of ordinary lignin derivatives (3–200 S/cm) owing to the larger size of the graphite flakes in LAG than ordinary lignin–graphite composites. The LAG size could be used to control the conductivity of the LAG-based films. The conduction and antiplasticizer effects of LAG for poly(ethylene glycol) (PEG) were also confirmed; that is, the mixing of LAG and PEG resulted in flexible conductive films without thermal annealing. Furthermore, the addition of LAG endows electroconductive properties to clay-based films with insulating characteristics. The preparation procedure of conducting materials does not require hazardous chemicals, such as surfactants and organic solvents, which have been used in the ordinary graphite dispersion process. The properties of SESC lignin as a dispersant of graphite that is free of other additives can be potentially useful in applications in various industrial fields.

## Materials and methods

### Materials

Ultrapure water was used throughout the study processes and was obtained using a Milli-Q^®^ Advantage A10^®^ system and Simplicity UV (Millipore™, Eschborn, Germany). Pyrolytic graphite powder (*φ* = 38 μm; PC99-300) and vein graphite powder (*φ* = 80 μm; ACB-100R) were purchased from Ito Graphite Co., Ltd. and Nippon Graphite Ind. Co., Ltd., respectively. Poly(ethylene glycol) (PEG; *M*_n_ = 500,000) was purchased from Wako Chemical Co., Ltd., and the clay mineral Li^+^ montmorillonite (MMT; Kunipia M by Kunimine Industries Co., Ltd., Japan) was used as received. The other reagent-grade chemicals were purchased from Tokyo Chemical Industry Co., Ltd. and DuPont™ Genencor^®^ Science and were used as received. The SESC lignin was prepared from Japanese cedar according to the basic procedures described elsewhere (Shikinaka et al., 2016).

For the SESC treatment, a mixture of cedar powder (10 w/w% for water; 0.01–2 mmφ), an enzyme cocktail prepared by combining equal amounts of commercial enzymes OPTIMASH XL containing cellulase and xylanase (10,300 U/g) and OPTIMASH BG containing xylanase and b-glucosidase (6,200 U/g) (DuPont™ Genencor^®^ Science), and about 100 mM of phosphate buffer (pH 6.0) was ground by bead milling (Labstar^®^ LMZ015; Ashizawa Finetech Ltd., Japan) at a peripheral velocity of 14.0 m/s at 50 °C. Stainless steel inactivates the enzyme; therefore, to prevent damage to the enzyme, the inner wall of the LMZ015 vessel was covered with a ceramic lining. Following bead milling for 2 h using 0.5 mmφ zirconia beads, the obtained mixture was centrifuged at 10,000×g for 30 min. The saccharide-containing supernatant was then collected, and the precipitate was milled once again under the same enzyme and buffer conditions but with 0.1 mmφ zirconia beads. The final slurry was centrifuged at 10,000×g for 30 min, following which the supernatant was recovered, and the lignin-rich precipitate (SESC lignin) was washed twice by mixing with equal amounts of ultrapure water before centrifugation under the aforementioned conditions. The SESC lignin was obtained as its water dispersion and drying this water dispersion produced the film or fine powder of SESC lignin (Shikinaka et al., 2016). The SESC lignin was extracted as nanoparticles of approximately several tens of nanometers diameter and amorphous shapes according to our previous work (Shikinaka et al., 2016; Shikinaka et al., 2018b). The purity of SESC lignin is 92% (Shikinaka et al., 2021), and it has nondeteriorated characteristics compared to other lignin derivatives (Shikinaka et al., 2016). The carbon content of SESC lignin is 56 wt% and was measured via elemental analysis by heating at 950 °C using the varioET III (Elementar Co., Germany).

### Preparation of LAG dispersed in water

For utilization as a coating film, about 2.5 g of graphite powder (PC99-300; >99% carbon content) was added to 50 ml of 2 wt% SESC lignin water dispersion. The mixture was then sonicated for 5 h at 400 W (Branson Sonifier 450, Emerson Japan, Ltd.). Until the end of sonication, room temperature was maintained by occasionally replacing the water in the sonicator bath. To remove the flakes of the nondispersed graphite powder, the mixture was centrifuged at 200–400×g for 10 min, and the supernatant was again centrifuged at 10,000 rpm for 30 min to remove the excess SESC lignin. Finally, the obtained slurry was sonicated for 1 h at 50 W (PR-1, Thinky, Japan) after adding an equal amount of pure water.

**FIGURE 1:**
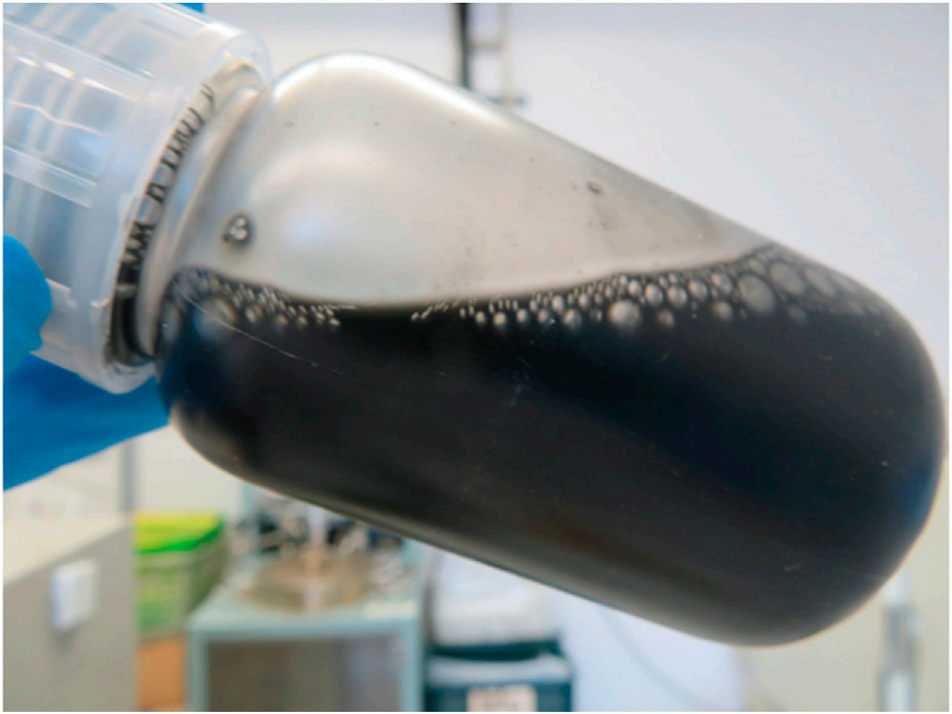
Photograph of typical graphite water dispersion obtained by sonication for SESC lignin water dispersion.

For utilization as a self-standing film, about 2.5 g of graphite powder (PC99-300 or ACB-100R; >99% carbon content) was added to 50 ml of 2 wt% SESC lignin water dispersion. The mixture was then sonicated for 5 h at 15 or 50 W (PR-1, Thinky, Japan). Until the end of sonication, room temperature was maintained by occasionally replacing the water in the sonicator bath. The mixture was centrifuged at 10,000 rpm for 30 min thereafter by adding pure water to remove the excess SESC lignin (ratio of graphite to SESC lignin in the obtained LAG is shown in the Results and discussion). Using the same procedure to yield LAG water dispersion, the obtained precipitate was diluted and centrifuged (Figure 1). To form a LAG film, 2 wt% PEG water solution was mixed with the LAG water dispersion at certain ratios. Here, LAG samples with various material compositions as shown in Table 1 were prepared to tune the properties of the LAG films.

**TABLE 1:** Preparation conditions of the LAG samples.

Sample name	Amount of LAG/wt%	Amount of PEG/wt%	Amount of MMT/wt%	Used graphite	Sonication power/W
Entry 1	100	0	0	PC99-100R	400
Entry 2	95	5	0	ACB-100R	15
Entry 3	95	5	0	PC99-300	15
Entry 4	95	5	0	ACB-100R	50
Entry 5	80	20	0	ACB-100R	15
Entry 6	50	50	0	ACB-100R	15
Entry 7	0	100	0	not used	not used
Entry 8	50	0	50	PC99-100R	15

### Preparation of LAG films

For preparation of the coated film for entry 1, LAG water dispersion was cast on a glass surface and dried in air at room temperature. For preparation of the self-standing films for entries 2–4, about 4 ml of the sample dispersion was dried by suction on a 100 nm cellulose acetate or carboxymethylcellulose filter (Merck Millipore) to mold the composite sheet. To obtain the self-standing films, several sheets were attached by pressing at 200 kN. The films were then hot pressed at 100 °C and 1,000 kN for 30 min, followed by annealing for 1 h at 300 °C.

For preparation of the self-standing films for entries 5–7, the sample dispersion was stirred for 2 h and then defoamed at 2,000 rpm for 10 min using a planetary centrifugal mixer (ARE-250 Thinly Co., Ltd., Japan). Casting and drying the obtained mixture produced the self-standing films, which were then hot pressed at 100 °C for 30 min. For preparation of the self-standing films for entry 8, a certain amount of MMT hydrogel was dispersed in ultrapure water in a homogenizer operated at 6,000 rpm for 15 min. The LAG water dispersion at a certain concentration was added to the MMT dispersion with stirring, which was continued for 25 min. The stirred mixture was cooled at room temperature for 30 min and again mixed at 2,000 rpm for 5 min using a planetary centrifugal mixer (ARE-250 Thinly Co., Ltd., Japan). Finally, the mixture was passed through a sieve with a 53 mm mesh and degassed at 2,200 rpm for 10 min using a planetary centrifugal mixer. The resulting gel-like mixture was cast onto a polyethylene terephthalate (PET) sheet using a film-casting knife (clearance gap of 0.6 mm) and dried under ambient conditions for approximately one day. The dried self-standing films were removed from the PET sheet to yield approximately 0.02 mm thick films. These films consisting of MMT were then thermally annealed for 10 h at 250 °C.

### Characterization of LAG and its films

Atomic force microscopy (AFM) observations were conducted using a Nanosurf FlexAFM. The samples for AFM were prepared by dropping the diluted dispersion onto freshly cleaved mica. Height profile analysis was then performed with Gwyddion (Nečas and Klapetek, 2012). Using a Rigaku Thermo plus EVO2 TG8120 system at a heating rate of 5 °C/min under normal air conditions, thermogravimetric (TG) analyses were conducted. X-ray photoelectron spectroscopy (XPS) measurements were additionally performed using the Ulvac Versa Probe II and Al-Kα radiation. The zeta potentials were finally measured with the ZEECOM ZC-3000 series (MICROTECH Co., Ltd.). All samples were tested three times, with 100 counts per measurement.

## Results and discussion

### Dispersing graphite in pure water by attaching SESC lignin

**FIGURE 2:**
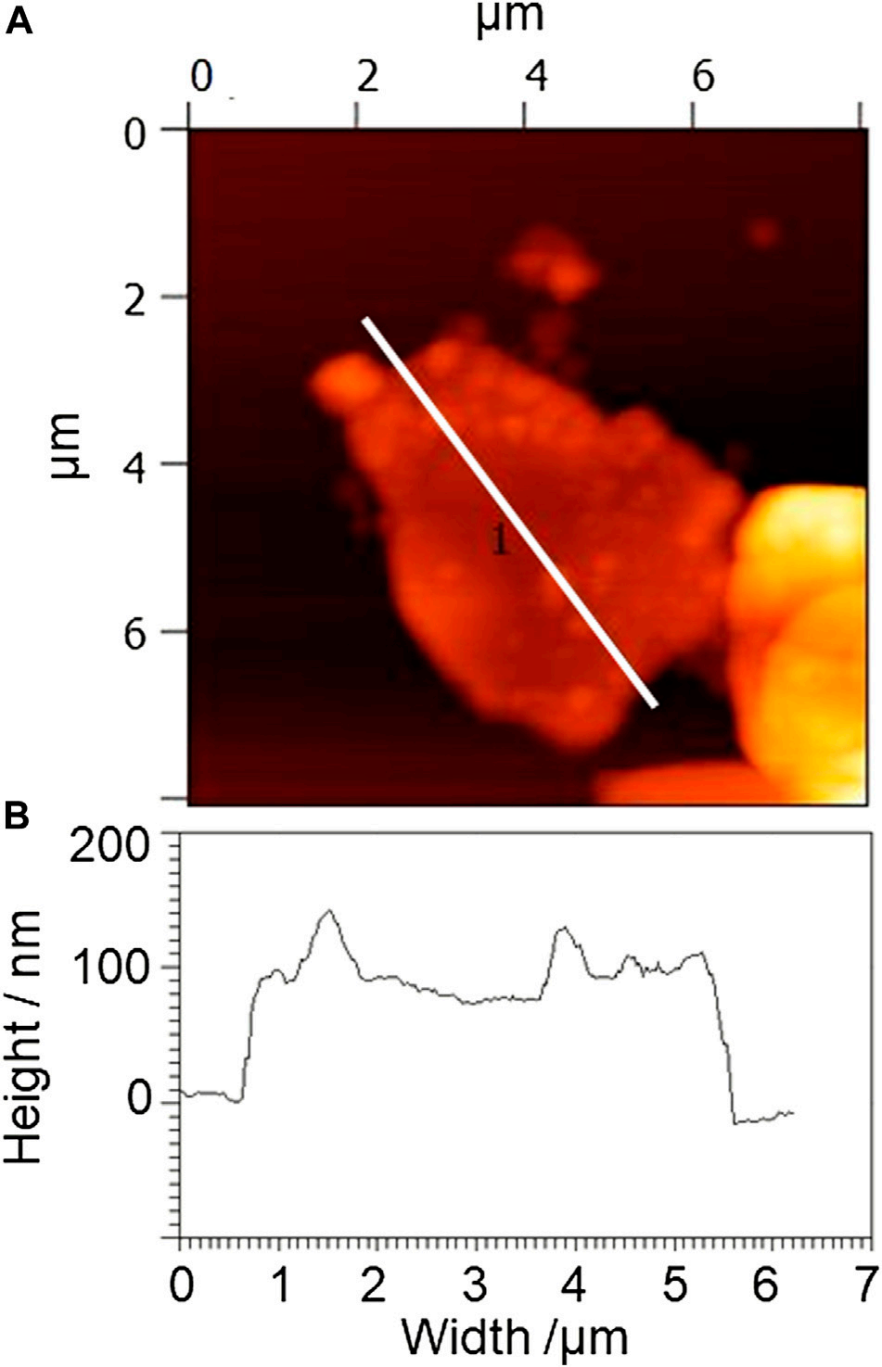
**(A)** Typical atomic force microscopy (AFM) image and **(B)** height profile of the white line in the AFM image of dried lignin-attached graphite (LAG) obtained by sonication at 15 or 50 W in graphite SESC lignin water dispersion. The red-colored amorphous flakes with an average diameter of several microns represent miniaturized graphite; the dark orange-colored particles on the flakes with an average diameter of several tens of nanometers are SESC lignin attached to the graphite.

Sonication of graphite powder and SESC lignin in pure water with 15 or 50 W power resulted in water dispersion (Figure 1) in which the graphite remained stably dispersed for several months. As shown in the AFM images of the dried dispersion (Figure 2), amorphous flakes with an average diameter of several microns and thickness of several hundred nanometers were observed, that is, sonication of graphite with SESC lignin in pure water causes graphite miniaturization and dispersion. Particles with an average diameter of several tens of nanometers were attached to these amorphous flakes; these particles have the same size order as the diameter of SESC lignin (Shikinaka et al., 2016), that is, the SESC-lignin-attached graphite flakes. Thus, sonication of graphite powder with SESC lignin in pure water resulted in water-dispersible LAG, i.e., SESC lignin acts as a dispersant for graphite that is free from toxic additives and organic solvents. Increasing the sonication power to 400 W results in thin LAG with miniaturized shape. However, exfoliation to a monolayer (e.g., graphene) was never observed in this experimental condition (Supplementary Figure S1).

A peak from the oxygen atom originating from the lignin molecules was observed in the XPS curve (Supplementary Figure S2). Additionally, oxidation of graphite was confirmed for the LAG sample, which has also been reported for lignin-sulfonate-attached graphene (Lou et al., 2015). These results indicate the presence of interactions between the SESC lignin and graphite, which causes SESC lignin adherence to graphite and dispersion of graphite in water. The sonication treatment for SESC lignin and graphite in pure water may induce an oxidation reaction between the graphite and SESC lignin. The peak positions in the X-ray diffraction (Supplementary Figure S3) and FT-IR spectra (Supplementary Figure S4) from LAG and neat graphite were almost similar.

The adhesion of several wt% of SESC lignin to graphite was confirmed after sonication with 15 W power by TG analysis (Supplementary Figure S5). The adhesion of the negatively charged SESC lignin (zeta potential: −102 mV) induces hydrophilicity of graphite, that is, the zeta potential of LAG in its solution is −90 mV. The LAG obtained by sonication of graphite and SESC lignin at 400 W showed adhesion of several tens of wt% of SESC lignin (Supplementary Figure S5). Increasing the sonication power for graphite dispersion thus facilitates adherence of SESC lignin to the surface of graphite because of further size miniaturization (e.g., increasing the surface area of graphite).

### LAG films with high electroconductivity

**FIGURE 3:**
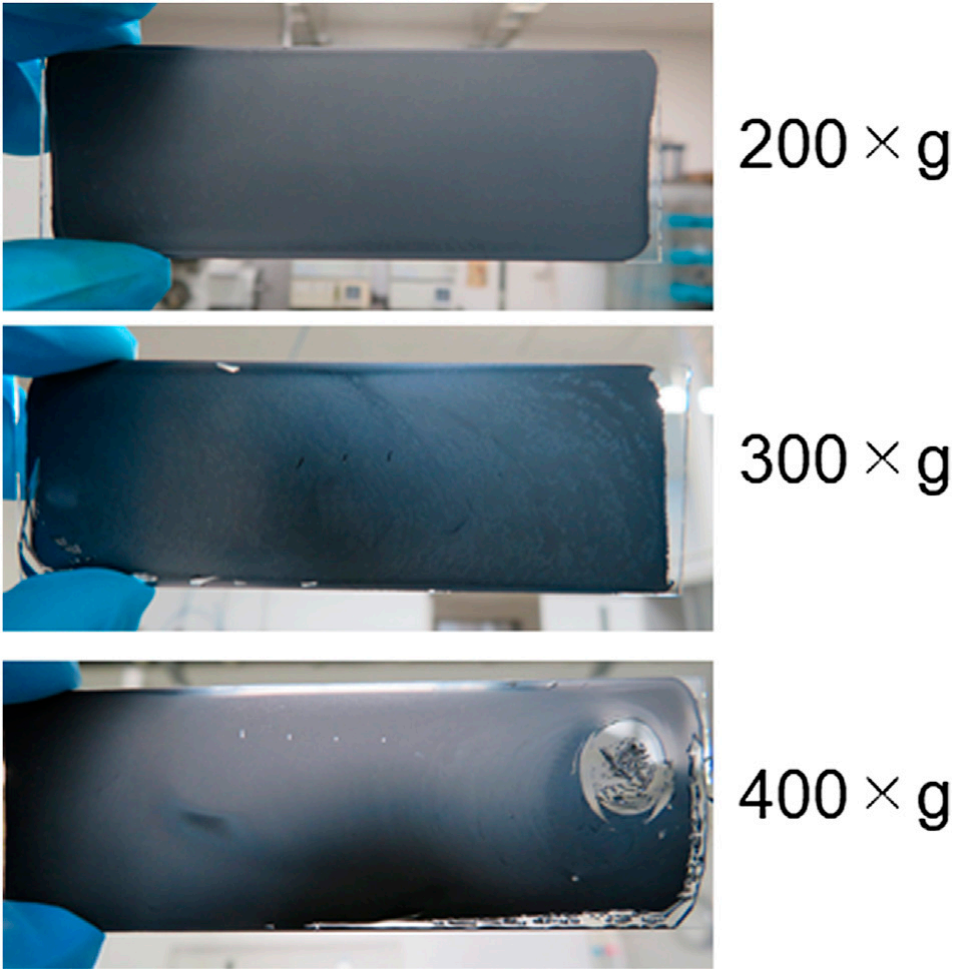
Photographs of typical coated LAG films on glass surfaces prepared at various gravity (×g) values for centrifugation to remove the flakes of nondispersed graphite powder from LAG dispersion.

Casting and drying LAG water dispersion on glass surfaces resulted in uniform coated films of LAG (Figure 3). Coating glass surfaces with LAG films induces electroconductive characteristics (Table 2), i.e., insulating property of glass is converted to conductive property by the LAG coating. The conductivity of LAG-coated glass increased with decreasing gravity of centrifugation to remove the flakes of nondispersed graphite powder (Table 2). The size of the remaining LAG in the dispersion should be larger at lower gravity of centrifugation. Thus, LAG water dispersion can be treated as an electroconductive ink.

**TABLE 2:** Relationship between conductivity and gravity for centrifugation to remove the flakes of nondispersed graphite powder from LAG dispersion for the LAG-coated films.

Sample name	Gravity for centrifugation/×g	Conductivity/S cm^−1^
Entry 1	200	497
Entry 1	300	175
Entry 1	400	38

**FIGURE 4:**
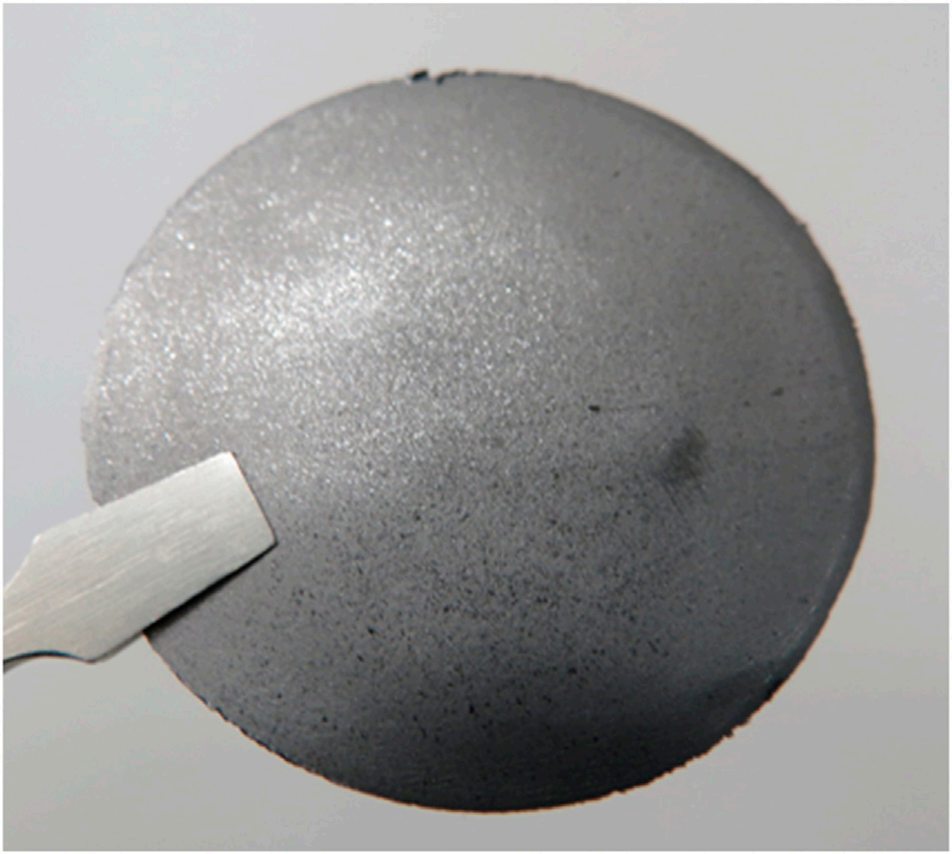
Photograph of a typical self-standing LAG film.

With the addition of PEG as a molding assistant, self-standing LAG films were obtained by drying and hot-pressing LAG (Figure 4). The self-standing LAG films have high conductivity, as shown in Table 3, even without thermal annealing. The PEG in the LAG films can be removed by annealing for 1 h at 300 °C (Supplementary Figure S3), which improves the conductivity of the LAG film. Here, LAG produced films with higher conductivity (2,075 S/cm) than graphene-based composites consisting of carbonized Kraft lignin (<210 S/cm; Follmer et al., 2019) or noncarbonized lignin sulfonate (<2.9 S/cm; Liu et al., 2019). Additionally, the LAG films can be obtained by thermal annealing at a lower temperature (300 °C) than graphene-based composites consisting of Kraft lignin (1,000–1,200 °C; Follmer et al., 2019).

**TABLE 3:** Relationship between LAG size and conductivity of the self-standing films.

Sample name	Particle size of LAG under SEM/μm	Conductivity before thermal annealing/S cm^−1^	Conductivity after thermal annealing/S cm^−1^
Entry 2	40	1482	2075
Entry 3	30	1067	1596
Entry 4	15	408	876

**FIGURE 5:**
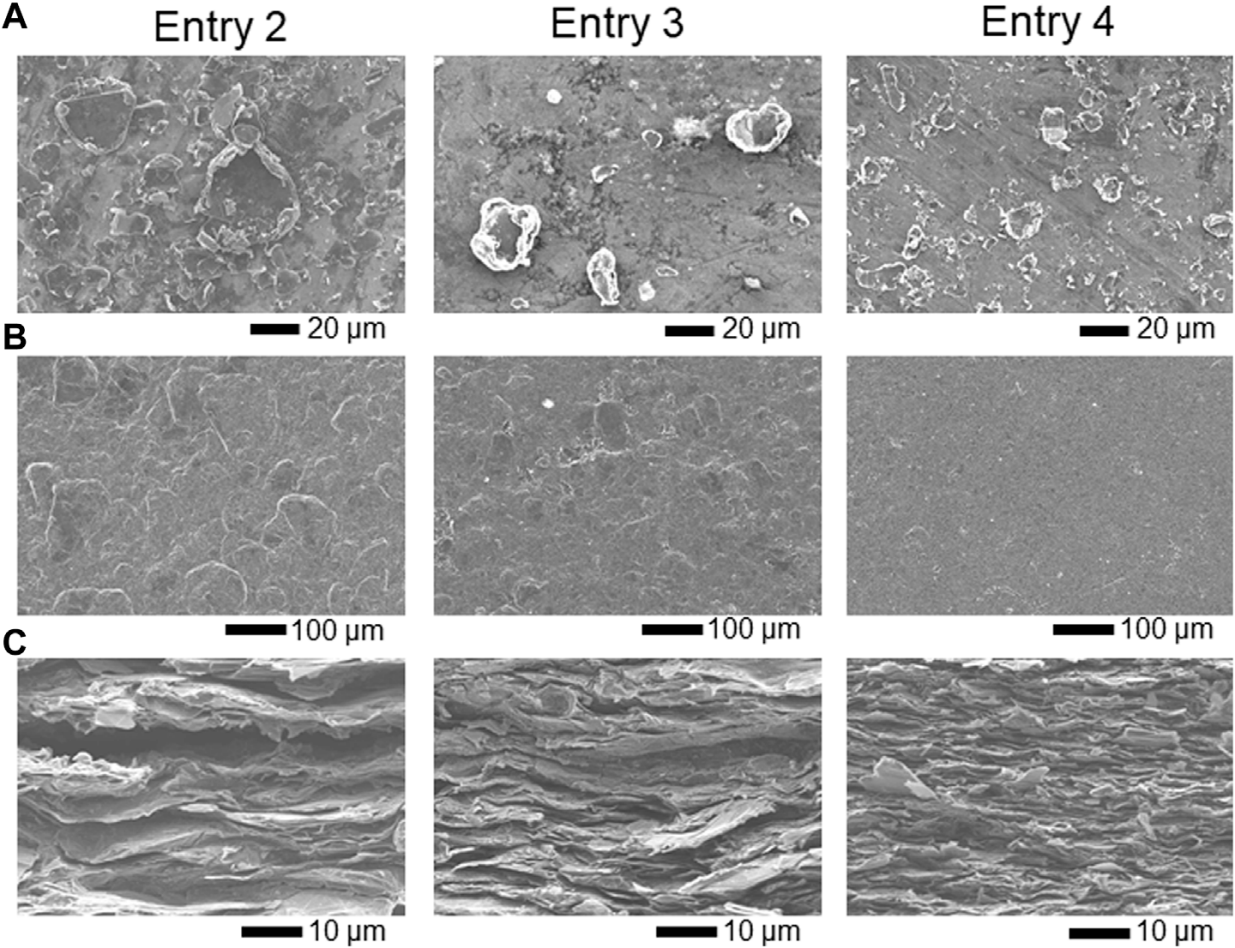
SEM) images of **(A)** LAG, **(B)** surfaces of the LAG films, and **(C)** cross sections of the LAG films for entries 1–3.

To estimate the relationship between the particle size and conductive properties of the LAG film, LAG with various particle sizes was prepared as per previous literature (Lin et al., 2012). As shown in Table 1, water-dispersed LAGs from different raw materials were obtained by sonication with 15 or 50 W power applied to the graphite–SESC-lignin water mixture. These procedures resulted in LAG with a particle size of several tens of microns (Figure 5A and Table 3). The particle size of the LAG decreased depending on the diameter of the raw graphite powder (as seen in entries 2 and 3) and sonication power (as seen in entries 3 and 4).

As shown in Table 3, the conductivity of the LAG film is proportional to the particle size of LAG, as observed by scanning electron microscopy (SEM). The SEM images of LAG films indicate that LAGs with smaller particle sizes have higher densities of LAG particles in the film (Figures 5B,C). Adding excess amounts (over several %) of SESC lignin to the polymer electrolyte adversely affects the ion conductivity of the electrolyte (Liu et al., 2022), i.e., structures of the SESC lignin and other lignin derivatives induce insulating characteristics in materials. Therefore, decreasing the area of the SESC-lignin-attached insulating interface on LAG that is inversely proportional to LAG size enhances the conductivity of the LAG film. In reality, the average size of the graphite flakes in LAG (15–40 μm) is much larger than that of the graphite flakes dispersed by noncarbonized lignin sulfonate (0.05–1 μm) (Liu et al., 2019), i.e., a small area of the lignin-attached insulating interface of graphite realizes high conductivity for graphite dispersed by SESC lignin. In the case of graphene, more attached points between the graphene flakes also affect the electroconductivity of the film (Nirmalraj et al., 2011). Thus, manufacturing self-standing films with micrometer-sized graphite flakes would result in LAG films with high electrical conductivity, i.e., SESC lignin enables the manufacturing of self-standing films of micrometer-sized graphite flakes that endows high electrical conductivity to the LAG film.

### Function as electroconductive filler of LAG for polymer films

**FIGURE 6:**
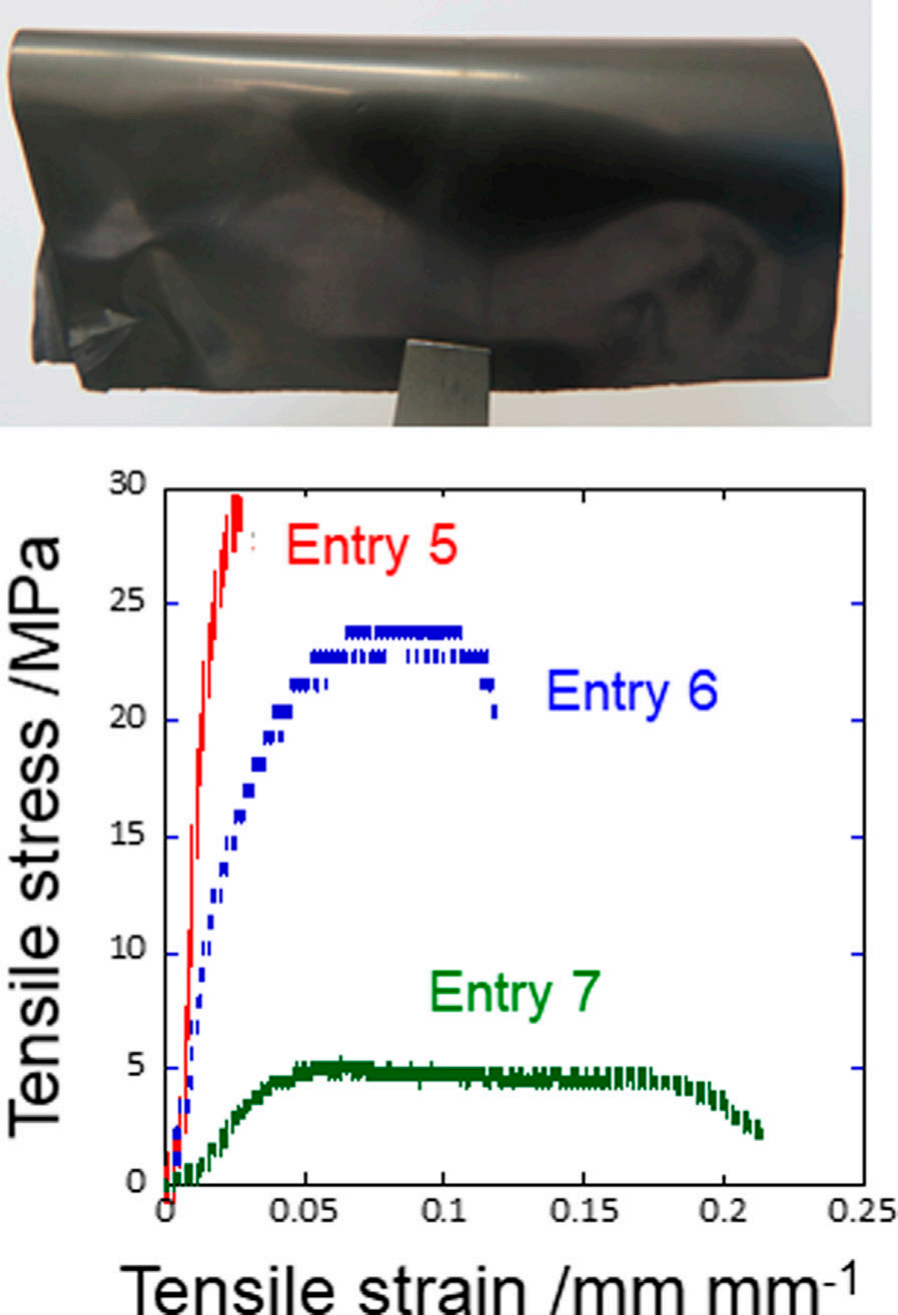
**(A)** Photograph of the typical LAG-PEG composite film and **(B)** its tensile stress–strain curves.

In pure water, LAG is finely mixed with an organic polymer such as PEG. Casting, drying, and hot pressing (not thermal annealing) the mixture of LAG and PEG resulted in a self-standing film (Figure 6). Adding LAG to PEG enhances the mechanical stiffness and electrical conductivity of the PEG film (Figure 6 and Table 4). Thus, LAG simultaneously acts as an antiplasticizer and a conductive filler for organic polymer films; hence, electrically conducting polymer films can be obtained through a water-based process without thermal annealing. Adding LAG to films consisting of a clay mineral like MMT that shows insulating properties also resulted in electroconductive properties, i.e., the volume resistivity of the MMT film dramatically decreased by ten orders of magnitude (MMT film without LAG: 1.4 × 10^9^ Ω cm (Suzuki et al., 2019), MMT film with 50 wt% LAG: 8.1 × 10^−1^ Ω cm).

**TABLE 4:** Mechanical properties of the LAG-PEG composite films.

Sample name	LAG/wt%	PEG/wt%	Conductivity/S cm^−1^	Fracture stress/MPa	Fracture strain/mm mm^−1^
Entry 5	80	20	155	21	3.4
Entry 6	50	50	3.30	22	10
Entry 7	0	100	N.D^a^	5.0	21

^a^ Too low conductivity to measure.

## Conclusion

Self-standing electrically conducting films were prepared using only graphite and lignin derivatives in this study *via* sonication in pure water without addition of other chemicals. The lignin derivative obtained by SESC facilitates dispersion of graphite in pure water through adhesion to the graphite surface under sonication treatment. Manufacturing self-standing films of micrometer-sized graphite using SESC lignin results in high electrical conductivity of the SESC LAG film. Furthermore, LAG simultaneously acts as an antiplasticizer and electroconductive filler for organic polymer films.

Electrically conducting films can thus be prepared using only graphite, SESC lignin, and pure water by an environmentally friendly process *via* purification of the lignin derivative, mixing of graphite with lignin, and film preparation without using hazardous chemicals. The film production described herein not only encourages utilization of plant-based components as high-value industrial materials but also reduces the environmental burden of extracting the limited petroleum-based resources.

## Data Availability

The original contributions presented in the study are included in the article/Supplementary Material, and further inquiries can be directed to the corresponding author.
