# Supplementary material for: Electrically conducting films prepared from graphite and lignin in pure water
Source: Front Bioeng Biotechnol. 2022 Nov 8;10:1049123. doi: 10.3389/fbioe.2022.1049123 (PMC9679407; doi:10.3389/fbioe.2022.1049123)
Supplement: Supplementary file 1 [file DataSheet1.docx]

Supplementary Material

# Supplementary Figures and Tables

## Supplementary Figures

**
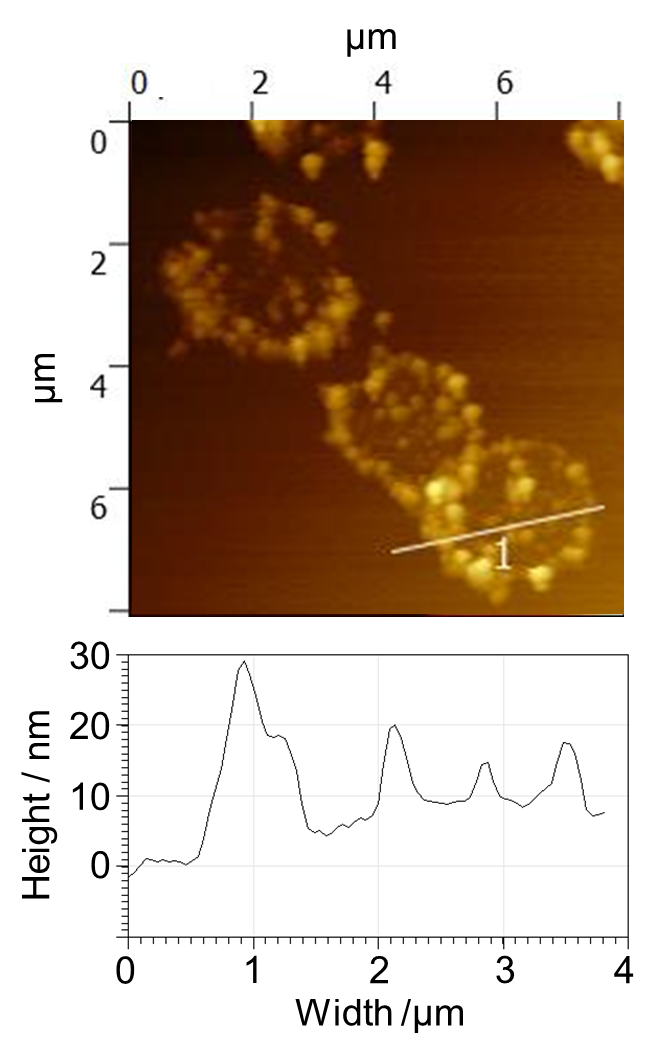
**

**Supplementary Figure 1.** Typical Atomic Force Microscopy (AFM) image and height profile of white line in AFM image of dried graphite dispersion prepared by sonication at 400 W power in the presence of simultaneous enzymic saccharification and comminution (SESC) lignin. Here 0.5 g of PC99-300 was dispersed in 49.5 mL of pure water. 53 mL of 4.8 wt% SESC lignin water dispersion was added little by little to the PC99-300 water dispersion under sonication at 400 W (Branson Sonifier 450, Emerson Japan, Ltd.). The mixture was stirred for 13 h and then centrifuged at 1,000 rpm for 10 min to remove flakes of non-dispersed graphite. To obtain graphite water dispersion, the supernatant was centrifuged at 10,000 rpm for 20 min to remove excess SESC lignin. The precipitate was dispersed in pure water by sonication for 2 h at 50 W (PR-1, Thinky, Japan) to obtain graphite water dispersion. From the height profile, the SESC lignin with a diameter of several tens of nm-attached graphite flakes was recognized. The thickness of miniaturized graphite is several nm, i.e., the graphite didn’t get exfoliated into a monolayer (graphene).


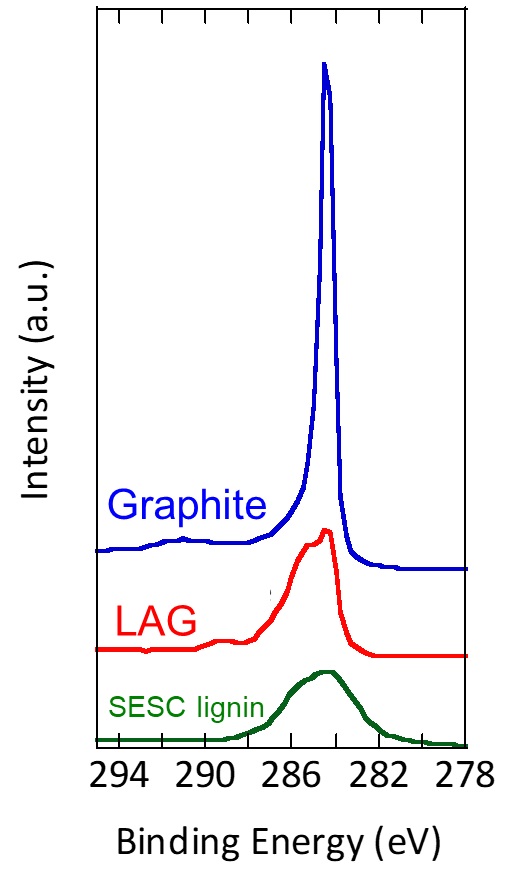

**Supplementary Figure 2.** (upper) High-resolution C_1S_ X-ray photoelectron spectra (XPS) for graphite (ACB-100R), simultaneous enzymic saccharification and comminution (SESC) lignin-attached graphite (LAG), and SESC lignin. (lower) Peak-separated XPS for graphite (left), LAG (middle), and SESC lignin (right). Graphite showed two peaks that originated from nonoxygenated ring C (284.4, 284,9 eV). SESC lignin has three peaks originating from nonoxygenated ring C (284.3 eV), the C in C-O bonds (286.0 eV), and the C in C-O-C bonds (287.8 eV). The LAG has four peaks that originate from nonoxygenated ring C (284.3 and 285.4 eV), the C in C-O bonds (286.4 eV), and the C in O-C=O bonds (289.1 eV) (Stankovich et al., 2006).S1 It seems that oxidation of graphite (that is, the emergence of the peak from the C in O-C=O bonds) occurred by sonication of graphite powder in the presence of SESC lignin that has been confirmed for lignin sulfonate-attached graphene (Lou et al., 2015).


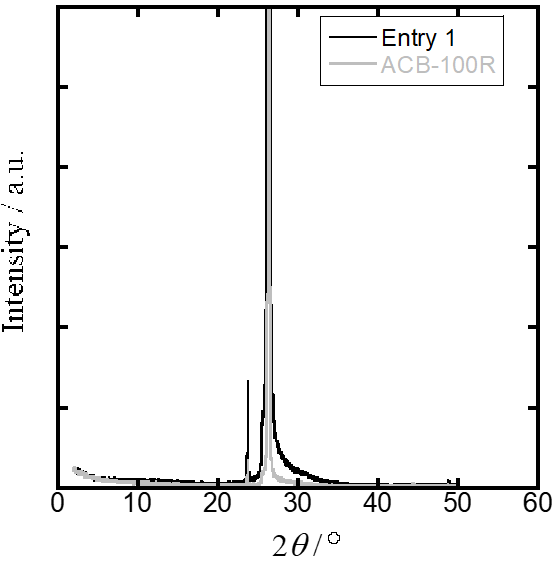


**Supplementary Figure 3.** Typical X-ray diffraction (XRD) pattern of LAG (Entry 1; black line) and graphite (ACB-100R; gray line). The XRD patterns were collected using a Rigaku Smart Lab X-ray diffractometer with Cu Kα radiation, a Cu Kβ filter, a 2.5° Soller slit, and a non-reflecting rotation stage. The collected count range was 2θ = 2–20° in 2θ increments of 0.02°.


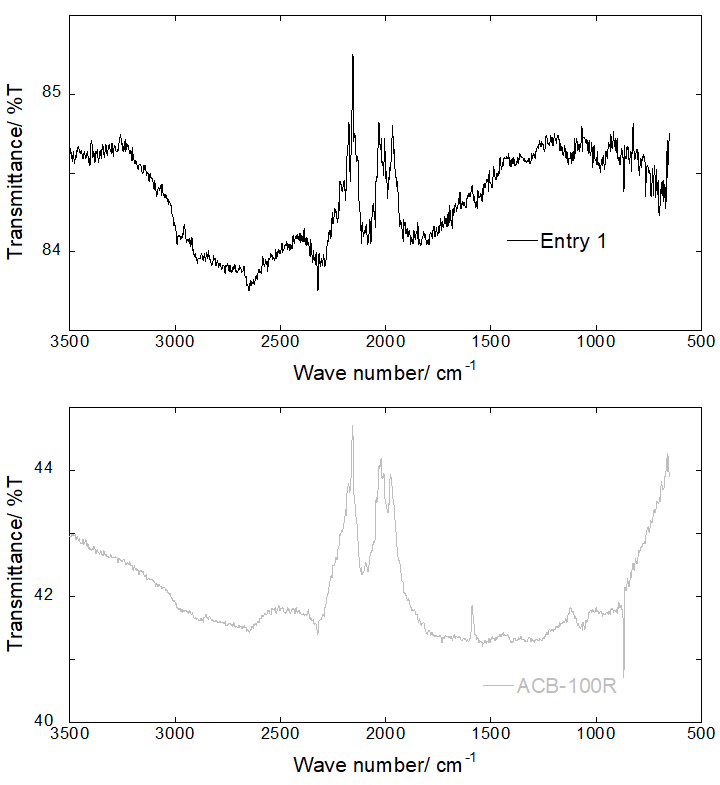


**Supplementary Figure 4.** Typical FT-IR spectrum of LAG (Entry 1) and graphite (ACB-100R). FT-IR spectra were recorded on a Thermo Sci NICOLET6700 spectrometer by attenuated total reflection method for sample powder.


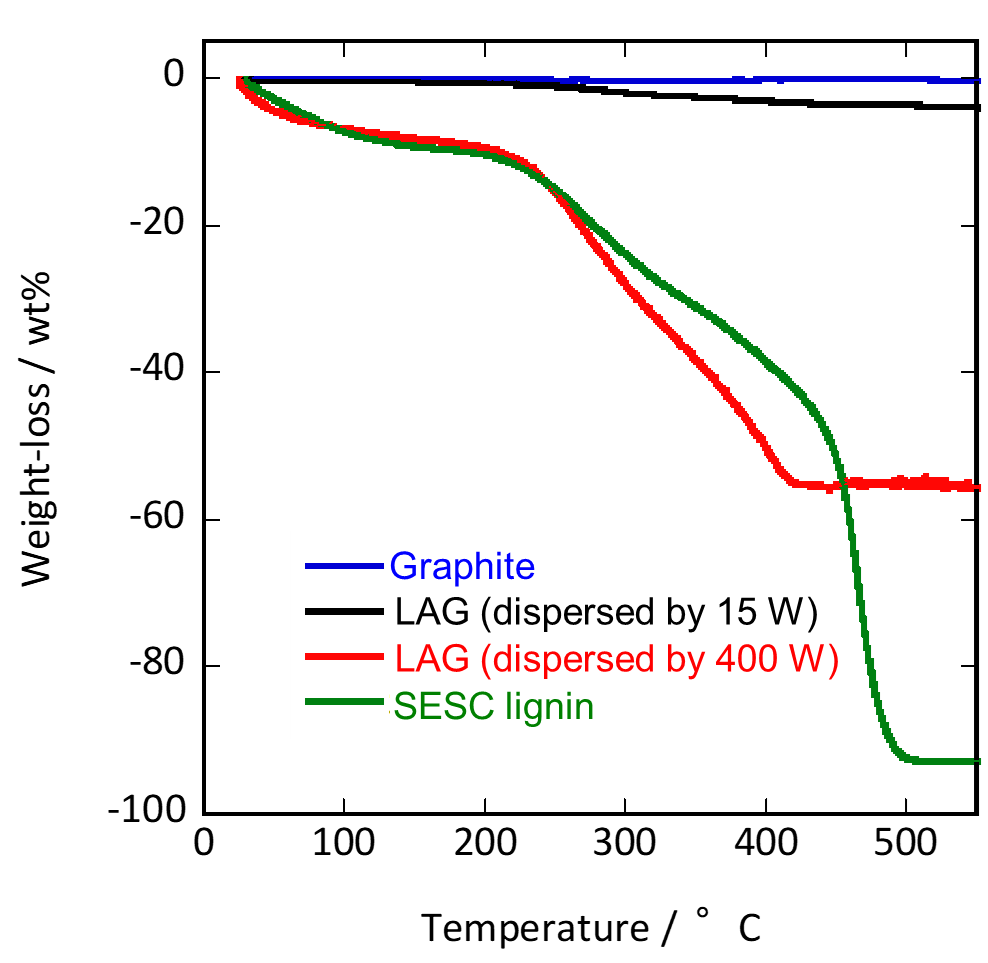


**Supplementary Figure 4.** (left) Thermogravimetric analysis (TGA) of graphite (ACB-100R), lignin-attached graphite (LAGs), and simultaneous enzymic saccharification and comminution (SESC) lignin. (right) TGA of LAG self-standing films (Entry 2) before or after heat annealing.

# References

Lou, H., Zhu, D., Yuan, L., Qiu, X., Lin, X., Yang, D.. and Li, Y. (2015). Fabrication of High-Concentration Aqueous Graphene Suspensions Dispersed by Sodium Lignosulfonate and Its Mechanism. *J. Phys. Chem. C* 119, 23221-23230. doi: 10.1021/acs.jpcc.5b06301

Stankovich, S., Piner, R. D., Chen, X., Wu, N., Nguyen, S. T., and Ruoff, R. S. (2006) Stable aqueous dispersions of graphitic nanoplatelets via the reduction of exfoliated graphite oxide in the presence of poly(sodium 4-styrenesulfonate). *J. Mater. Chem.* 16, 155-158. doi: 10.1039/b512799h
